# Maltose and maltotriose utilisation by group I strains of the hybrid lager yeast *Saccharomyces pastorianus*

**DOI:** 10.1093/femsyr/fow053

**Published:** 2016-06-30

**Authors:** Frederico Magalhães, Virve Vidgren, Laura Ruohonen, Brian Gibson

**Affiliations:** 1 VTT Technical Research Centre of Finland Ltd, Tietotie 2, PO Box 1000, FI-02044 VTT, Espoo, Finland; 2 Department of Biotechnology and Chemical Technology, Aalto University, School of Chemical Technology, Kemistintie 1, PO Box 16100, FI-00076 Aalto, Espoo, Finland

**Keywords:** Group I, lager, hybrid, maltotriose, α-glucoside transporters, *MTT1*

## Abstract

Brewer's wort is a challenging environment for yeast as it contains predominantly α-glucoside sugars. There exist two subgroups of the lager yeast *Saccharomyces pastorianus* which differ in sugar utilisation. We performed wort fermentations and compared representative strains from both groups with respect to their ability to transport and ferment maltose and maltotriose. Additionally, we mapped the transporters *MALx1*, *AGT1*, *MPHx* and *MTT1* by Southern blotting. Contrary to previous observations, group I comprises a diverse set of strains, with varying ability to transport and ferment maltotriose. Of the eight group I strains, three efficiently utilised maltotriose, a property enabled by the presence of transmembrane transporters *SeAGT1* and *MTT1*. A58, a variant of the group I type strain (CBS1513) performed particularly well, taking up maltotriose at a higher rate than maltose and retaining significant transport activity at temperatures as low as 0°C. Analysis of transporter distribution in this strain revealed an increased copy number of the *MTT1* gene, which encodes the only permease known with higher affinity for maltotriose than maltose and low temperature dependence for transport. We propose that much of the variation in lager yeast fermentation behaviour is determined by the presence or absence of specific transmembrane transporters.

## Abstract (precis)

Maltotriose transport and fermentation by group I *Saccharomyces pastorianus* strains.

## INTRODUCTION

Brewing yeasts are divided into two classes, ale yeast and lager yeast, according to their industrial use. Ale beer is brewed by domesticated strains of *Saccharomyces cerevisiae* at relatively high temperatures (15°C–26°C), while lager beer is traditionally brewed by the yeast *S. pastorianus* at lower temperatures (5°C–15°C). *Saccharomyces pastorianus* is now known to be a hybrid yeast resulting from a cross between *S. cerevisiae* (probably an ale strain) and the cold-tolerant yeast *S. eubayanus* (Dunn and Sherlock 2008; Libkind *et al.*^2011^). Two genetically distinct lineages exist within the *S. pastorianus* taxon, group I (or Saaz) and group II (or Frohberg) (Liti *et al.*^2005^; Dunn and Sherlock 2008). The historical distinction between the groups was partly based on differential utilisation of sugars in brewer's wort, particularly the inability of group I strains to use maltotriose (Morris 1895; Glendinning 1899; Johnson 1904).

The terms Saaz and Frohberg are not encountered in the brewing literature of the latter half of the century. Group I yeast were gradually replaced by group II strains in industrial breweries during the 20th century, probably due to the superior fermentation performance of the latter. However, the group I strain CBS1513 (still commonly referred to by the traditional designation *S. carlsbergensis*) has remained in use at the Carlsberg brewery since 1883 (Walther, Hesselbart and Wendland 2014), suggesting an efficient fermentation performance in this strain, possibly related to α-glucoside utilisation.

The two groups differ in terms of DNA content; the Carlsberg strain contains 47 chromosomes in a roughly triploid genome (3n-1) and group II strain Weihenstephan 34/70 has 64 chromosomes (4n) (Nakao *et al.*^2009^; Walther, Hesselbart and Wendland 2014). Different theories on how these two groups originated have been proposed. Conserved translocations between CBS1513 (synonym *S. carlsbergensis*; group I) and Weihenstephan 34/70 (group II) suggest a common origin, while differences in DNA content and chromosome copy number suggest independent hybridisation events (Dunn and Sherlock 2008; Walther, Hesselbart and Wendland 2014). The distribution of so-called ‘lager specific’ genes in subtelomeric regions supports a model of separate hybridisation events, involving different classes of ale strains (Monerawela *et al.*^2015^). In a recent study, the analysis of genome sequences of 10 lager strains suggests that both groups share at least one common hybridisation event, and that differences in ploidy may be due to chromosomal loss in group I strains or that a second hybridisation with a *S. cerevisiae* strain may have given rise to the group II lineage (Okuno *et al.*^2016^). These genetic differences appear to impact on fermentation performance of the two groups. Group I strains seem better adapted to ferment at lower temperatures (Gibson *et al.*^2013^; Walther, Hesselbart and Wendland 2014) but typically lack the ability to use maltotriose (Gibson *et al.*^2013^).

The utilisation of maltotriose is of major importance in brewing as it represents ca. 20% of the fermentable sugars in brewer's wort, maltose being the most abundant sugar at ca. 60% and sucrose, glucose and fructose together representing the remaining 20%. Glucose and fructose are readily fermented by the yeast as they are transported into the cell by facilitated diffusion, and glucose represses the synthesis of maltose and maltotriose transporters and maltases and inactivates pre-existing transporters (Federoff, Eccleshall and Marmur 1983; Goldenthal *et al.*^1987^; Lucero, Herweijer and Lagunas 1993; Jespersen *et al.*^1999^; Maier *et al.*^2002^; Rautio and Londesborough 2003). After the hexoses are used, maltose is the preferred sugar, and maltotriose is typically the most abundant sugar in the later stages of fermentation.

There are three different transporters known to carry maltose, Mal, Agt1 and Mtt1 (Jespersen *et al.*^1999^; Salema-Oom *et al.*^2005^; Vidgren, Ruohonen and Londesborough 2005). Due to the hybrid nature of the lager yeast, some of the transporters were inherited from both parents and exist in two allelic forms, the *S. cerevisiae* and *S. eubayanus* counterparts. There are five unlinked *MAL* loci (*MAL1-4* and *MAL6*). In the classical *MAL* locus, there are three genes; *MALx1* (where *x* denotes the locus of origin, 1–4 or 6) encodes a transporter, *MALx2*, an α-glucosidase and *MALx3*, a transcriptional activator of the other two genes. All *MALx1* transporter genes share high identity (>95%) and are generally regarded as very specific high-affinity maltose transporters (Km, 2–5 mM), without transport activity for maltotriose (Chang *et al.*^1989^; Dietvorst, Londesborough and Steensma 2005).

Agt1 (α-glucoside transporter), encoded by a naturally occurring allele of *MAL11* (*MAL11g*) with 57% identity to *MAL11*, is a relatively wide-spectrum α-glucoside transporter, with higher affinity for trehalose and sucrose (Km 8 mM); lower affinity for maltose (Km 5–17.8 mM), maltotriose (Km 18.1 mM) and α-methylglucoside (Km, 20–35 mM); and the lowest affinity for isomaltose, melezitose and palatinose (Han *et al.*^1995^; Stambuk *et al.*^1999^).

In all lager strains tested by Vidgren, Ruohonen and Londesborough (2005) and Vidgren *et al.* (2009), the *AGT1* gene derived from the *S. cerevisiae* parent (*ScAGT1*) was found to have a premature stop codon at nucleotide 1183 and to encode a non-functional 394 amino-acid polypeptide, while the ale strains encoded a full-length 610 amino-acid protein. Genome sequencing of the lager strain *S. pastorianus* Weihenstephan 34/70 revealed the presence of another gene located in the left subtelomere of the non-*S. cerevisiae* chromosomes XV with 79% identity to the *S. cerevisiae AGT1* (Nakao *et al.*^2009^). This was thought to be a homologue from the non-*S. cerevisiae* parent of the lager yeast (Nakao *et al.*^2009^). This transporter, presumed to be *S. eubayanus AGT1* (*SeAGT1*), was further characterised by Vidgren and Londesborough (2012), who demonstrated that, despite having only 85% identity at amino-acid level with ScAgt1, it has also a broad substrate specificity with similar affinities for maltose and maltotriose (Km, 17 and 22 mM, respectively).

Finally, *MTT1* (*MTY1*) was identified by screening genomic libraries of lager strains (Dietvorst, Londesborough and Steensma 2005; Salema-Oom *et al.*^2005^). This transporter shares 90% identity with *MALx1* and 54% with *ScAGT1* genes. It transports maltose, maltotriose, trehalose and turanose, with the unusual ability, among α-glucoside transporters, to carry maltotriose with higher affinity than maltose (Km of 16–27 mM for maltotriose and 61–88 mM for maltose), a trait which is particularly relevant in brewing (Dietvorst, Londesborough and Steensma 2005; Salema-Oom *et al.*^2005^).

In addition to these, there are two identical transporter genes, *MPH2* and *MPH3*, thought to be involved in maltose utilisation. The role of these transporters has been questioned with different studies reporting contradictory results in terms of transport, gene expression and inconsistent correlation between the presence of these transporters and α-glucoside utilisation phenotypes (Jespersen *et al.*^1999^; Vidgren, Ruohonen and Londesborough 2005; Alves *et al.*^2008^; Duval *et al.*^2010^). Brown, Murray and Verstrepen (2010) showed that these transporters are only able to transport turanose; nevertheless, we decided to include it in our study to confirm their inability to utilise α-glucoside.

Sugar utilisation and, consequently, fermentation performance among lager strains vary greatly. In this study, we investigated the relationship between the presence of certain α-glucoside transporters and fermentation performance of a set of brewing yeast strains including lager and ale strains, as well as the lager parent strain *S. eubayanus*. Special emphasis was given to lager yeast group I as little was known about maltotriose utilisation by these strains. In addition, we performed electrophoretic karyotyping and mapped the maltose/maltotriose transporter genes by Southern blotting. Although a modern genome sequencing approach could be employed for these kind of studies, difficulties in sequencing of subtelomeric regions, where sugar transporters are localised, combined with the complex nature of hybrid genomes makes our approach more feasible for studying a large number of strains. These data allowed us to determine the role of specific transporters in maltotriose utilisation during fermentation and discover how certain strains maintain efficient sugar transport at low temperatures.

## MATERIALS AND METHODS

### Materials

All-malt wort was prepared at VTT Technical Research Centre of Finland. Worts were prepared with Espoo city water (all other water was deionised and filtered through active carbon using the MilliQ Water System; Millipore Corporation, MA). Worts were collected hot (>90°C) and stored at 0°C until use. Stored worts were mixed before use to resuspend the settled solids evenly. A 15 ºP wort contains 20.0 g L^−1^ glucose, 5.6 g L^−1^ fructose, 67.2 g L^−1^ maltose and 24.7 g L^−1^ maltotriose. [U-^14^C]-maltose (ARC 488) and [U-^14^C]-maltotriose (ARC 627) were from American Radiolabeled Chemicals (St. Louis, MO, USA). [U-^14^C]-maltotriose was repurified before use as described by Dietvorst, Londesborough and Steensma (2005). Maltose (minimum purity, 99%) and maltotriose (minimum purity, 95%) were from Sigma-Aldrich (St. Louis). ^32^P-γ-ATP was from Perkin Elmer (Turku, Finland).

### Yeast strains

The strains used in this work were obtained from VTT culture collection (http://culturecollection.vtt.fi/) and are summarised in Table 1. This list includes a variant of the Carlsberg strain CBS1513 deposited at VTT culture collection in 1974 with the code A-74058. Abbreviated codes are used in the text here, as indicated between brackets in Table 1. Strains were maintained in agar-solidified YP (10 g L^−1^ yeast extract and 20 g L^−1^ peptone) containing 40 g L^−1^ of maltose.

**Table 1.** List of *Saccharomyces* strains used in this study.

Strain reference			
VTT	Other relevant codes	Strain group	Observations
A-61011 (A11)		I	
A-60012 (A12)		I	
A-78053 (A53)		I	
A-74058 (A58)		I	Isolate of *S. carlsbergensis* type strain deposited at VTT culture collection in 1974
A-14231 (A231)	CBS1513, GSY129	I	Former *S. carlsbergensis* type strain
A-06203T (A203)	CBS1503, GSY134	I	Former *S. monacensis* type strain
A-13221 (A221)	GSY133	I	
A-13222^T^ (A222)	CBS1538, DBVPG6047, GSY131	I	Saaz type strain
A-63015 (A15)		II	Finnish lager yeast strain
A-13220 (A220)	Weihenstephan 34/70	II	
A-75060 (A60)		Ale	Finnish ale yeast strain
A-93115 (A115)	NCYC 1063	Ale	
C-12902 (C902)	CBS12357	*S. eubayanus*	Type strain

### Group I and group II differentiation

The group of each strain was confirmed as described in Pham *et al.* (2011). The ribosomal RNA-encoding DNA (rDNA) internal transcribed spacer (ITS) region was amplified by PCR using the primers ITS1 (5^′^-TCCGTAGGTGAACCTGCGG-3^′^) and ITS4 (5^′^-TCCTCCGCTTATTGATATGC-3^′^). The product was digested with the restriction enzyme HaeIII (New England Biolabs, Hitchin, UK) yielding a three-band pattern (490, 225, 140 bp) for the group I strains and four bands (320, 225, 180, 140 bp) for the group II.

### Tall tube (static) fermentation

Brewer's wort fermentation was carried out essentially as previously described (Rautio and Londesborough 2003; Gibson *et al.*^2013^) with the use of a ‘G0 fermentation’ prior to the actual experimental fermentations to reflect the fact that most industrial brewery fermentations are started with recycled rather than freshly propagated yeast. Approximately 2 L of 15°P wort was fermented with selected strains in stainless steel cylindroconical vessels (so-called tall tubes) at a pitching rate of 5 g of fresh yeast L^−1^. The fermentations were carried out at 15°C until no change in the residual extract was observed for 24 h.

Samples were withdrawn daily, centrifuged at 9000 rpm for 10 min at 1°C and the supernatant was used for wort/beer analyses. The pellet was washed with water, weighed, resuspended in water and used for yeast analyses. Dry yeast masses were determined by drying the washed yeast slurry overnight at 105°C in a pre-weighed porcelain crucible.

### Wort and beer analyses

The specific gravity, alcohol level and pH of samples were determined from the centrifuged and degassed fermentation samples using an Anton Paar density meter DMA 5000 M with Alcolyzer Beer ME and pH ME modules (Anton Paar GmbH, Austria).

Concentration of fermentable sugars (glucose, fructose, maltose and maltotriose) was measured by HPLC using a waters 2695 separation module and waters system interface module liquid chromatograph coupled with a waters 2414 differential refractometer (Waters Co., Milford, MA, USA). An Aminex HPX-87H organic acid analysis column (300 × 7.8 mm, Bio-Rad) was equilibrated with 5 mM H_2_SO_4_ (Titrisol, Merck, Germany) in water at 55°C, and samples were eluted with 5 mM H_2_SO_4_ in water at a 0.3 mL min^−1^ flow rate.

### Maltose and maltotriose transport assays

For maltose and maltotriose uptake measurement, the yeast strains were grown at 20°C in liquid YP medium containing maltose (4% w/v) to an OD600 nm between 4 and 8. The cells were harvested by centrifugation (10 min, 5000 rpm, 0°C), washed with ice-cold water and 0.1 M tartrate-Tris (pH 4.2) and resuspended in the same buffer to a concentration of 200 mg fresh yeast mL^−1^. Zero-trans rates of [U-^14^C]-maltose and [U-^14^C]-maltotriose uptake at 20°C, 10°C or 0°C were determined with 5 mM of substrate as described by Lucero *et al.* (1997). Two incubation times were tested to ensure linearity with respect to time with t2 corresponding to at least 90% of t1 value. Statistical analysis was performed with R (http://www.rproject.org/; v3.3.0) by using one-way ANOVA and Tukey's test.

### Quantitative PCR for copy number determination

The copy numbers of the transporter genes *ScMALx1*, *SeMALx1*, *ScAGT1*, *SeAGT1*, *MPHx* and *MTT1* were estimated with quantitative PCR of genomic DNA. DNA was extracted from the strains using a GeneJET Genomic DNA Purification kit (Thermo Scientific, USA) with an additional cell disruption step using acid-washed glass beads (Sigma-Aldrich, Finland). The plasmid pUG66 was used as an internal standard (Gueldener *et al.*^2002^). The primers were designed using PerlPrimer (version 1.1.21). The PCRs were performed using a LightCycler^®^ 480 SYBR Green I Master mix (Roche Diagnostics, Switzerland) on a LightCycler 480 II instrument (Roche Diagnostics) with four independent replicates. The following program was used: pre-incubation (95°C for 5 min), amplification cycle repeated 45 times (95°C for 10 s, 60°C for 10 s, 72°C for 10 s with a single fluorescence measurement), melting curve program (65°C–97°C with continuous fluorescence measurement) and finally a cooling step to 40°C. The efficiencies (*E*) of the qPCR assays (ranging from 1.96 to 2.00) for each primer pair were calculated using the formula 10^(−1/m)^, where m is the slope of the line of the threshold cycle (*C_T_*)-versus-log dilution plot of the DNA template (5 pg to 50 ng input DNA; Pfaffl 2001). The raw data analysis was performed using LinRegPCR (version 2016.0; Ruijter *et al.*^2009^). The relative copy numbers of the target genes were calculated using the formula (*E*_target_)*^Δ^^CT^*^,target (control-sample)^/ (*E*_reference_)*^Δ^^CT^*^,reference (control-sample)^ (Pfaffl 2001). To reduce the error caused by possible variation of the initial genomic DNA concentration, the results were adjusted by dividing by a dilution factor calculated based on the assumption that each strain has a single copy of *ScAGT1*.

### Chromosome separation by pulse field gel electrophoresis (PFGE)

Sample plugs were prepared using CHEF Yeast Genomic DNA Plug Kit (Bio-Rad, Helsinki, Finland) according to the manufacturer's instructions. Plugs were loaded into the wells of a 1% Pulse Field Certified Agarose (Bio-Rad, Helsinki) gel. PFGE was performed at 14°C in 0.5 × TBE buffer (89 mM Tris, 89 mM boric acid, 2 mM EDTA [pH 8]). A CHEF Mapper XA Pulsed Field Electrophoresis system (Bio-Rad) was used with the following settings: 6 V cm^–1^ at a 120° angle, pulse length increasing linearly from 26 to 228 s and total running time of 38 h. A commercial chromosome marker from *Saccharomyces cerevisiae* strain YNN295 (Bio-Rad, Helsinki) was used as molecular mass reference. After electrophoresis, the gels were stained with ethidium bromide and visualised with Gel Doc XR+ system (Bio-Rad).

### Chromosome blotting and hybridisation

Chromosomes separated by PFGE were partially depurinised by soaking the gels in 0.25 M HCl for 10 min, denatured for 2 × 15 min in 0.5 M NaOH–1.5 M NaCl and finally neutralised 2 × 15 min in 1.5 M NaCl–0.5 M Tris buffer (pH7.5). The DNA was transferred to a Nylon membrane (Hybond-N; GE Healthcare Life Sciences) by capillary blotting in 20 × SSC (3 M NaCl–0.3 M sodium citrate [pH 7.0]). After blotting, the DNA was UV cross-linked to the membrane using UV stratalinker 2400 (Stratagen, Kirkland, WA, USA) in auto cross-link mode. Prehybridisation was carried out at 37°C for 2 h in a solution containing 6 × SSPE (0.9 M NaCl, 0.06 M sodium phosphate, 0.03M EDTA [pH 7.4]), 10 × Denhardt solution (0.2% BSA, 0.2% Polyvinylpyrrolidone K30, 0.2% Ficoll 400), 0.5% SDS and 100 μg of denatured herring sperm DNA. The membranes were then soaked in a hybridisation mix containing 6 × SSPE, 0.1% SDS and 10–15^6^ CPM of labelled oligonucleotide probe and incubated overnight at 50°C. The filters were finally washed twice with 2 × SSC–0.05% SDS at room temperature for 5 min and in 0.1 × SSC–0.1% SDS for 20 min at 45°C. Filters were exposed for 2 days to a phosphor imager screen and visualised using GE Healthcare's Typhoon Trio Variable Mode Imager. The probes were removed by washing the filters with 0.1 × SSC–0.1% SDS for 20 min at 80°C and the filters were reprobed two more times. The bands detected were associated with the respective chromosome based on the size relative to the molecular weight marker and reference strains A220, A231 and A203 as described in van den Broek *et al.* (2015).

### Probe labelling and purification

The oligonucleotide probes (Table S1, Supporting Information) were obtained from Integrated DNA Technologies and labelled by phosphorylation of the 5^′^ hydroxyl terminus of the oligonucleotides using ^32^P-γ-ATP with DNA 5’ end labelling kit MEGALABEL (Takara, Tokio, Japan). Labelled probes were purified by gel filtration with illustra NAP-5 Columns (GE Healthcare, Helsinki, Finland) and eluted in 1 × TE buffer (10 mM Tris-HCl, 1 mM EDTA).

## RESULTS

### Confirmation of group status

Thirteen strains including the *Saccharomyces eubayanus* type strain, two ale strains, two group II lager strains and eight group I lager strains were selected for investigation. The group of each lager strain was confirmed by ITS amplification and digestion with HaeIII according to the method of Pham *et al.* (2011) and matched previous classifications, when these were available. Group I strains yielded the characteristic three band profile and the group II strains a four band pattern (Fig. S1, Supporting Information).

### **Fermentation of 15**°**P wort**

**Figure 1.**
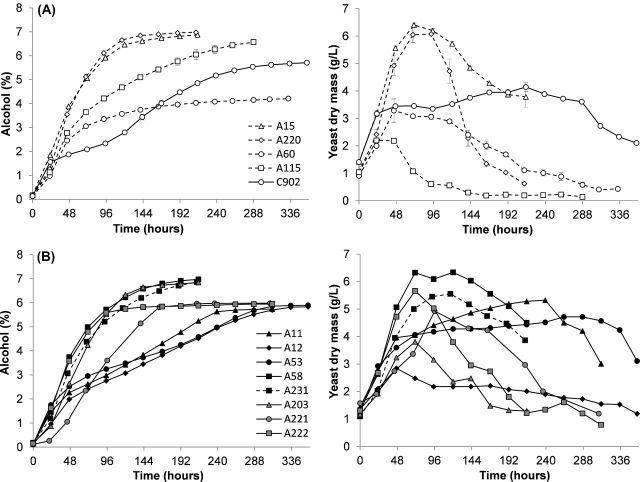
Fermentation (%ABV) of 15°P all-malt wort at 15°C and suspended yeast dry mass (g L^−1^). Panel **(A)** includes group II, ale and *S. eubayanus* strains; panel **(B)** includes group I strains. Values are means from two independent fermentations and error bars where visible represent the range.

The two group II strains efficiently fermented the wort to 6.9%–7% alcohol by volume (ABV) after 215 h (Fig. 1A) corresponding to 81%–83% apparent attenuation. The ale strains performed differently, with A115 reaching 6.6% ABV after 288 h while A60 was unable to exceed 4.2% ABV even after 336 h (Fig. 1A) with 77% and 49% apparent attenuation, respectively. *Saccharomyces eubayanus* C902 fermented the wort to 5.7% ABV (Fig. 1A) and 67% apparent attenuation. Interestingly, we observed here two different profiles among the group I strains. While the fermentation profiles of five strains (A11, A12, A53, A221 and A222) are in line with the work of Gibson *et al.* (2013) and ceased fermentation after reaching 5.7%–6% ABV and 69%–70% apparent attenuation, the performance of strains A58, A231 (CBS1513) and A203 resembles the fermentation performances of the group II strains with alcohol levels in the range of 6.8%–7.0% ABV and 80%–83% apparent attenuation after 215 h (Fig. 1).

Analysis of the remaining sugars at the end of the fermentation revealed differential utilisation of maltotriose*. Saccharomyces eubayanus* and most of the group I strains were not able to utilise maltotriose at all (Table 2). There was again variation in group I as three of the strains, A231, its variant A58 and A203, utilised maltotriose as efficiently as group II strains. Results between two ale strains differed and this was probably because the A60 strain stopped fermenting earlier, leaving 47% of maltose and 83% of maltotriose unfermented. This might be due to the low temperature used (15°C) which is not optimal for an ale strain. In general, when alcohol level did not exceed 6% at the end of the fermentation, all initial maltotriose still remained unused in the wort (except for the ale strain A60).

**Table 2.** Concentration of the α-glucosides maltose and maltotriose at the end of the fermentation. Values are in g L^−1^ and represent the mean of two independent fermentations ± standard deviation.

	Strain	Maltose (g L^−1^)	Maltotriose (g L^−1^)
	15°P Wort	67.2 (±2.28)	24.7 (±1.12)
Group I	A11	n.d.	24.7 (±0.89)
	A12	n.d.	24.6 (±0.78)
	A53	n.d.	24.7 (±0.88)
	A58	n.d.	1.9 (±0.76)
	A231	5.8 (±0.28)	2.7 (±0.02)
	A203	n.d.	6.5 (±0.91)
	A221	n.d.	24.4 (±0.93)
	A222	n.d.	24.7 (±0.97)
Group II	A15	n.d.	6.2 (±0.72)
	A220	n.d.	2.9 (±0.10)
Ale	A60	31.7 (±0.7)	20.6 (±0.34)
	A115	n.d.	6.6 (±0.18)
*S. eubayanus*	C902	n.d.	24.7 (±0.77)

n.d. – not detected.

The yeast mass in suspension was similar in both group II strains, reaching its peak of >6 g L^−1^ at 71 h (Fig. 1A). The relatively low yeast mass values observed in the ale strains are consistent with the poor fermentation of the A60 strain and the highly flocculent character of A115 (data not shown). Once again, the group I strains displayed higher variability in terms of yeast mass with no consistent relationship with fermentation performance, although the faster fermenting strains A58, A222 and A231 did reach the highest amount of yeast mass in suspension (Fig. 1B).

**Figure 2.**
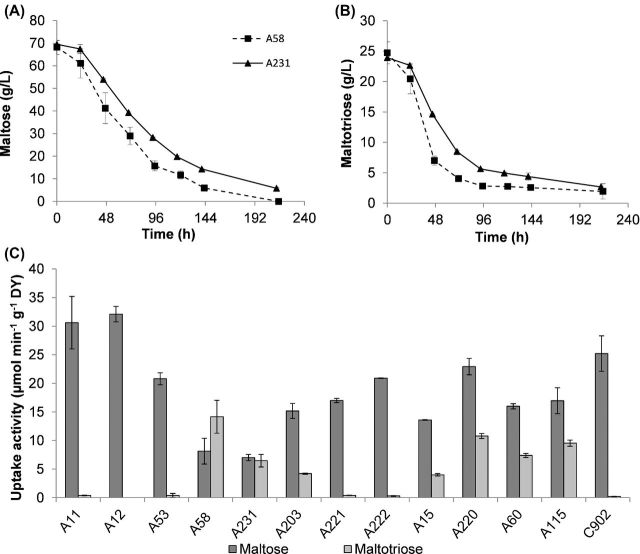
Time profile of (**A**) maltose and (**B**) maltotriose concentration (g L^−1^) of group I strains A58 and A231 throughout the fermentation. Values represent the mean of two independent fermentations and error bars the standard deviation. (**C**) Zero-trans rates of maltose and maltotriose uptake activity (μmol min^−1^ g^−1^ DY) of the strains in study, measured at 20°C. An uptake activity ≤0.5 μmol min^−1^ g^−1^ DY is considered negligible. Values are means of three independent assays, error bars represent standard deviation.

Due to the close relationship between A231 (CBS1513) and its variant strain A58, the time profile of sugar consumption was examined in more detail (Fig. 2A and B). Both strains show co-consumption of maltose and maltotriose. A58 is particularly adept at utilising maltotriose with 72% reduction of maltotriose concentration after 48h of fermentation. At the same time point, A231 had utilised only 39%. However, there was some residual maltotriose left at the end of the fermentation period by both strains (Fig. 2B, Table 2). The exception in maltose utilisation among lager strains was A231 (CBS1513) which did not completely use maltose; 5.8 (±0.28) g L^−1^ were still detected at the end of the fermentation (Fig. 2A, Table 2).

### Maltose and maltotriose uptake by the cell

To characterise the α-glucoside transport properties of strains, the uptake activity of maltose and maltotriose was quantified. Maltose uptake values in strains studied varied in the range of 7.0 to 32.1 μmol min^−1^ g^−1^ DY. Higher activity did not necessarily ensure faster fermentation. In fact, strains with highest maltose uptake activity (Fig. 2C) were relatively slow fermenters (A11, A12, A53 and C902; Fig. 1). Results show clearly that maltose transport capacity alone is not sufficient to explain differences in fermentation performance.

Maltotriose transport activity was detected only in those strains able to utilise this sugar during fermentation. The group I strains A11, A12, A53, A221 and A222 and *S. eubayanus* C902 had negligible transport activity for maltotriose (<0.5 μmol min^−1^ g^−1^ DY; Fig. 2C, Table 2). All group II and ale strains a well as the group I strains A58, A231 and A203 had transport activities of 4.0–14.2 μmol min^−1^ g^−1^ DY, which is sufficient to support utilisation of maltotriose (Fig. 2C, Table 2). The group I strain A231 (CBS1513) and its variant A58 displayed particularly high maltotriose uptake rates relative to maltose uptake, with A231 having approximately the same transport activity for both maltose and maltotriose, and A58 with 75% more activity toward maltotriose than for maltose. The A58 variant was also the strain with the highest rate of maltotriose uptake among the tested strains and with the lowest residual maltotriose by the end of fermentation (Fig. 2C, Table 2). This unusual phenotype of lager yeast, i.e. more efficient maltotriose uptake compared to maltose, has never been described before; it does however fit with the fermentation properties observed for this strain (Fig. 1; Table 2). A number of mostly maltotriose-utilising group I strains was selected for further investigation.

### Effect of temperature on overall transport activity

Sensitivity of transport activity to low temperature was found to be variable, with clear differences between the ale and lager groups. Lager strains retained 45%–58% of the relative maltose transport activity at 10°C (except A11 strain with specific maltose/maltotriose transporter genotype, see below/discussion), which is significantly higher than in ale strains at about 30% (*P* < 0.01; Table 3). At 0°C, maltose transport activity of lager strains was still significantly different from ale strains (8%–20% versus 3%; *P* < 0.05). Similarly for maltotriose uptake, lager strains retained 34%–50% of transport activity at 10°C, whereas ale strains retained only 6%–18% (*P* < 0.05). At 0°C, corresponding values were 3%–13% compared to 1%–2% in lager and ale strains, respectively. The *S. eubayanus* strain lost more transport activity than lager strains at 10°C and activity was at the level of ale strains at this temperature. However, between 10°C and 0°C temperature sensitivity of *S. eubayanus* was levelling off and at 0°C *S*. *eubayanus* retained the same amount of activity as lager strains in general. Among the lager strains, there is still no direct relationship observed between strain group and the cold sensitivity of maltose transport. For the uptake of maltotriose, however, apparently higher activity was retained among the group I strains, however not statistically different (*P* > 0.05). Interestingly, one strain (the CBS1513 variant A58) retained 13% of its activity at 0°C, enough to support uptake of maltotriose (i.e. higher than 0.5 μmol min^−1^ g^−1^ DY).

**Table 3.** Relative maltose and maltotriose transport activity retained when the assay temperature is reduced to 10°C or 0°C. Values represent the mean percentage (%) activity relative to values at 20°C % ± standard deviation of three independent experiments.

		Maltose		Maltotriose	
Group	Strain	10°C	0°C	10°C	0°C
Group I	A11	24 (±1.8)	9 (±0.8)	n.d.	n.d.
	A58	45 (±6.6)	11 (±3.1)	34 (±6.0)	13 (±2.9)
	A231	46 (±3.8)	8 (±0.4)	49 (±8.4)	9 (±1.3)
	A203	49 (±2.1)	20 (±0.9)	50 (±0.7)	7 (±0.1)
Group II	WS 34/70	50 (±2.7)	13 (±0.4)	34 (±1.9)	3 (±0.2)
	A15	58 (±0.8)	16 (±2.3)	43 (±2.6)	8 (±0.2)
Ale	A60	29 (±0.5)	3 (±0.1)	6 (±0.3)	2 (±0.1)
	A115	30 (±2.0)	3 (±0.3)	18 (±1.2)	1 (±0.0)
*S. eubayanus*	C902	33 (±2.4)	15 (±1.2)	n.d.	n.d.

n.d. – Not determined; strains that have no significant activity at 20°C.

### Detection and quantification of transporter genes by Southern hybridisation and qPCR

Among the strains studied, different phenotypes were observed for properties relevant for beer fermentation, e.g. the rapid utilisation of maltotriose and cold tolerance of the maltose and maltotriose uptake. To relate these observations with the genotypes of the strains, the chromosomes were separated by PFGE (a representative gel is shown in Fig. S2, Supporting Information), blotted onto a nylon membrane and hybridised with specific probes that allow not only differentiation between each transporter gene (*MALx1*, *AGT1*, *MPHx* and *MTT1*) but also the origin of each transporter (*S. cerevisiae* or *S. eubayanus*) (Fig. S3–S8, Supporting Information). Due to the high identity (57%–95%) between the maltose and maltotriose transporters, we used short probes of 31–40 nucleotides to be able to find unique sequences not hybridising to other maltose/maltotriose transporter genes. The probes were sensitive enough to discriminate between transporters of different origin, e.g. *S. cerevisiae* probes did not hybridise to *S. eubayanus* chromosomes and no *S. eubayanus* transporter genes were found in the ale strains. Weaker chromosome bands for the strain A11 in the PFGE may affect the intensity of the bands detected by Southern blot; however, to validate these data, the relative copy number of each transporter gene was assessed by qPCR.

The genotypes of the strains in study are described in Table 4 and Table S3 (Supporting Information). Distribution of *ScMALx1* loci varied a lot between the strains (Fig. S3, Supporting Information). The A58 strain had only one *MALx1* locus, *MAL41*, A231 two loci, *MAL11* and *MAL41*, whereas all other strains had three to four *MALx1* loci.

**Table 4.** α-Glucoside transporter genotypes of the studied strains.

	Transporter genes					
Strain	*ScMALx1* ^a^	*SeMALx1* ^b^	*ScAGT1* ^a^	*SeAGT1* ^b^	*MPHx* ^a^	*MTT1*
A11	II (*MAL31*), XI (*MAL41*), XIII	II-IV, XI, SeVII-ScVII	–	–	–	–
A58	XI (*MAL41*)	II-IV, SeVII-ScVII, XV-XVII, V	VII	XV-VIII	–	II, SeIII-ScIII, VI, VII, ScVIII, SeXI
A231	VII (*MAL11*), XI (*MAL41*)	II-IV, SeVII-ScVII, XV-XVII, V	VII	XV-VIII	–	VI, IX, SeXI
A203	II (*MAL31*), VII (*MAL11*), XI (*MAL41*)	II-IV, SeVII-ScVII	VII	>XV-VII	–	VI, VII, SeXI
A15	II (*MAL31*), VII (*MAL11*), XI (*MAL41*)	II-IV, XI, SeVII-ScVII, IX	VII	XV-VIII	IV (*MPH2*)	SeIII-ScIII, VII
A220	II (*MAL31*), VII (*MAL11*), VIII (*MAL61*), XI (*MAL41*)	II-IV, XI, SeVII-ScVII, IX	VII	XV-VIII	IV (*MPH2*)	SeIII-ScIII, VII
A60	II (*MAL31*), III (*MAL21*), VII (*MAL11*), XI (*MAL41*),	–	VII	–	–	–
A115	II (*MAL31*), III (*MAL21*), XI (*MAL41*)	–	VII	–	IV (*MPH2*)	VII
C902	–	XI, VII, XV	–	–	–	–

a *S. cerevisiae* chromosomes where the probe hybridised.

b *S. eubayanus* chromosomes where the probe hybridised.

Three to four loci of *S. eubayanus MALx1* were found in the lager strains (Fig. S4, Supporting Information). However, the role of this transporter remains uncertain. In lager yeast, *SeMALx1* genes are most probably non-functional pseudogenes as *SeMaLx1* genes cloned from lager strains WS34/70 and A15 were shown to be truncated and non-functional (own non-published results). Also, based on whole genome sequencing of WS34/70, there are no full-length open reading frames present in this strain able to encode a functional protein (Nakao *et al.*^2009^).

*ScAGT1* was detected on the *S. cerevisiae* chromosome VII of all lager and ale strains investigated, except A11 (Fig. S5, Supporting Information). Faint bands were detected in smaller chromosomes of lager strains with this probe; however, these were not considered to be *ScAGT1* based on known distribution of this transporter genes in representative strains (i.e. A220 and A15; Vidgren, Ruohonen and Londesborough 2005; Nakao *et al.*^2009^) and the fact that strain A11 is unable to take up maltotriose suggests that these genes do not encode a functional transporter. As shown in earlier studies, ScAgt1 is non-functional in lager strains due to frame shift mutation, whereas in ale strains Agt1 is functional and the only transporter known to carry maltotriose (Vidgren, Ruohonen and Londesborough 2005).

The probe specific for the *S. eubayanus* allele of the *AGT1* gene hybridised to a chromosome of about 750 kb possibly corresponding to *S. eubayanus* hybrid chromosome XV–VIII (Fig. S6, Supporting Information). Among the six lager strains included here, only A11 was apparently missing *SeAGT1*, contrarily amplification was observed by qPCR but the lack of maltotriose transport activity supports the absence of an active SeAgt1 transporter in A11. In A203, it was detected on a larger chromosome (∼800 kb). It was however not detected in the *S. eubayanus* strain or in the ale strains.

The *MPH2* transporter was found on chromosome IV of the group II strains and in the ale strain A115 (Fig. S7, Supporting Information). In strain A220, a second band was detected in a chromosome of approximately 1125 kb, possibly chromosome VII. The identical *MPH3* (chromosome X) was only detected in the *S. cerevisiae* strain YNN295, used for molecular size calibration (Fig. S7, Supporting Information).

There was a lot of variation also in the *MTT1* locus distribution among the strains (Fig. S8, Supporting Information). In the group II strains, two alleles were found on chromosomes III and VII, however in the group I strains the distribution of this gene seems to be more variable; it was missing in A11, three copies were found in A231 and A203 both of which share a copy at chromosome VI and SeXI, but the former has a second copy at chromosome IX and the latter at chromosome VII. In the A58 strain however the *MTT1* probe mapped to six different chromosomes that can be tentatively identified as II, SeIII-ScIII (*S. cerevisiae*: *S. eubayanus* hybrid chromosome III), VI, VII ScVIII and SeXI. The *MTT1* gene was also found to be present at chromosome VII of the A115 ale strain but missing in A60 and *S. eubayanus* C902. Overall, the Southern hybridisation data was backed up by the qPCR analysis, however variations may be caused by the fact that the data obtained by the two techniques are not necessarily the same, situations when a gene is duplicated in the same chromosome or different chromosomes of the same size cannot be identified. The combination of these techniques allows for an accurate determination of copy number and allocation to its position in the genome (Tables 4 and S3, Supporting Information).

## DISCUSSION

The transport of maltose into the cell has been suggested to limit the rate of the fermentation (Kodama *et al.*^1995^; Meneses and Jiranek 2002; Rautio and Londesborough 2003). Similarly, efficient transport of maltotriose is essential for its depletion from brewer's wort (Zheng *et al.*^1994^; Stambuk and de Araujo 2001; Gibson *et al.*^2013^; Krogerus *et al.*^2015^). The limit of fermentation performance therefore appears to be defined by a strain's capacity to transport both maltose and maltotriose.

Differential use of maltotriose has been a defining feature of group I and group II strains for more than a century (Morris 1895; Glendinning 1899) and a recent study supports this observation (Gibson *et al.*^2013^). However, here we show that group I comprises a phenotypically diverse set of strains with differential use of maltotriose. In this study, three of eight strains in group I had an efficiently fermenting, maltotriose-utilising phenotype, while four had a non-maltotriose-utilising, slowly fermenting phenotype. In addition, a third phenotype was observed for the strain A222, efficiently attenuating the wort to 67% in 96 h but being unable to ferment further due to its inability to use maltotriose. These results contradict observations of Duval *et al.* (2010), who classified this strain (referred to as DBVPG6047 in that study) as an efficient maltose and maltotriose-utilising strain. Nevertheless, the ability of the group I strains A231 and A203 to use maltotriose was reported in the same study and is in line with our observations.

Temperature dependence of single maltose/maltotriose transporters has been shown to decrease in the following order ScAgt1(from ale) > ScMalx1(lager) > SeAgt1(lager) > Mtt1(lager) (Vidgren *et al.*^2010^, ^2014^). These temperature dependences are in agreement with the results of this study, as strains possessing SeAgt1 and Mtt1 transporters were shown to retain more activity at low temperature. This is especially evident when comparing ale and lager strains. Lager strains maintain relatively high transport activity at low temperatures and possess *SeAGT1* and several copies of *MTT1* gene (except the A11 strain) compared to ale strains (which are missing *SeAGT1* and have only one copy of *MTT1* gene at most). Strain A11 was the exception within the lager strain group chosen for detailed analysis, as it contained only *MALx1* type genes and lacked genes related to good cold tolerance in α-glucoside transport. This genotype was associated with less efficient maltose uptake activity at low temperatures compared to other lager strains. It was also shown that the ale strain A115, which possesses *MTT1*, retained more maltotriose transport activity at 10°C compared to the A60 ale strain which does not possess this gene. The only α-glucoside transporter gene found to be present in the *Saccharomyces eubayanus* strain, *SeMALx1*, has not been fully characterised and thus the temperature dependence of this single transporter in relation to other maltose/maltotriose transporters is not known. However, as it is the only maltose/maltotriose transporter present in the *S. eubayanus* strain (based on hybridisation results) it must be the only one carrying maltose. At 10°C, *S. eubayanus* had lost much of its relative maltose transport activity and activity was similar to that of ale strains. This is unexpected since *S. eubayanus* is a cold-tolerant strain and has been shown to grow well on maltose at low temperatures (Gibson *et al.*^2013^). However, between 10°C and 0°C *S. eubayanus* does not lose as much activity as ale strains and activity is actually at the same level as lager strains at 0°C. The advantage conferred by the hybrid nature of the lager strains was particularly evident here; clearly higher relative uptake activities at low temperature were observed compared to the ale strains and even to *S. eubayanus.*

In group I strains, there were significant differences in uptake activities, e.g. the A58 strain carried maltotriose more efficiently than maltose, A231 had similar transport activity for these two sugars and the A11 strain was not able to carry maltotriose at all. The distribution of transporters in these strains indicated that variations in uptake were caused by different distributions of *MAL* and *MTT1* genes. This is because *SeMALx1* genes are thought to be non-functional pseudogenes in lager strains (based, e.g., on whole-genome sequence analysis; Nakao *et al.*^2009^), the *ScAGT1* gene is non-functional in lager strains (Vidgren, Ruohonen and Londesborough 2005) and *MPH* genes are absent; thus, none of these genes can affect overall transport activity. Moreover, as *SeAGT1* is similarly present in all strains able to carry maltotriose it cannot be the cause of these differences. *ScMALx1* and *MTT1* gene distribution correlated well with maltose and maltotriose uptake properties. In the strain A58, which carried maltotriose more efficiently than maltose, there was only one copy of the *ScMALx1* locus and six copies of *MTT1.* In the A231 strain, which takes up maltose and maltotriose equally efficiently, the number of *ScMALx1* loci was higher at two and the apparent copy number of *MTT1* was lower at three. In the A203 strain, which used maltose more efficiently than maltotriose, the number of *MALx1* loci was three whereas *MTT1* copy number remained at two.

The origin of Mtt1 is still a matter of debate, this transporter was initially identified by screening genomic libraries of lager strains (Dietvorst, Londesborough and Steensma 2005; Salema-Oom *et al.*^2005^), it was later detected in both *S. cerevisiae* and *S. eubayanus*-type chromosome VII of the Weihenstephan 34/70 genome (Nakao *et al.*^2009^) and it has been generally hypothesised to be of *S. eubayanus* origin (Dietvorst, Londesborough and Steensma 2005; Nakao *et al.*^2009^; Vidgren *et al.*^2010^; Cousseau *et al.*^2013^). Here we found that *MTT1* was distributed among several chromosomes, the identification of the origin of the chromosome is however not simple since the high number of chromosomes in these strains results in overlapping bands in the PFGE.

Interestingly, we found *MTT1* in the ale strain A115. This calls into question the hypothesised *S. eubayanus* origin. Vidgren *et al.* (2010) also found it to be present in baker's and distiller's *S. cerevisiae* strains. The currently limited availability of *S. eubayanus* strains hampers the study of maltose transporters in this species. So far only the Patagonian isolate has been characterised to any extent. However, strains have been isolated in North America (Peris *et al.*^2014^), China (Bing *et al.*^2014^) and recently in New Zealand (Gayevskiy and Goddard 2016). Studying maltotriose utilisation in these strains may help elucidate the origin of the lager yeast transporters. Similarly to *MTT1*, the origin of *SeAGT1* has also been questioned based on higher sequence similarity of both transporter sequences to genes in *S. cerevisiae* than in *S. eubayanus*. This suggests that both genes may be inherited from the *S. cerevisiae* parent of the lager yeast, i.e. are paralogues rather than orthologues (Baker *et al.*^2015^). None of these genes here was detected in *S. eubayanus* C902, instead only the *SeMALx1* probe hybridised to three different chromosomes, V, VII and XVI. These results are however not unexpected based on the inability of this strain to use maltotriose.

Mphx transporters were not found in any of the group I strains tested; this is consistent with observations that the presence or absence of *MPHx* transporters does not directly relate to maltotriose utilisation (Duval *et al.*^2010^). *MPH2* was however found in the group II strains and in the ale strain A115. Given the subtelomeric location of the transporters, these results support the theory of separate hybridisation events involving different *S. cerevisiae* lineages raised by Monerawela *et al.* (2015).

The differences here observed between the Carlsberg strain (A231) and its variant (A58) are likely to reflect variations within a population of the same strain. Such variation is not unprecedented; within the WS34/70 population, variant strains have been described with differences in flocculation, attenuation and flavour profile (Bolat, Walsh and Turtoi 2008). The high copy number of *MTT1* alleles in A58 likely gave this clonal line an advantage in brewing conditions, particularly if it was exposed to particularly low temperatures.

In conclusion, we show that maltotriose transport activity is essential for the utilisation of this α-glucoside and that *MTT1* plays an essential role in uptake of this sugar by lager strains, particularly at the low temperatures employed in lager fermentation. We propose that much of the variation in the fermentation behaviour that is known to exist within the lager brewing yeast is determined by the presence or absence of specific transmembrane transporters.

## Supplementary Material

Supplementary Data
