# Supplementary material for: Maltose and maltotriose utilisation by group I strains of the hybrid lager yeast Saccharomyces pastorianus
Source: FEMS Yeast Res. 2016 Jun 30;16(5):fow053. doi: 10.1093/femsyr/fow053 (PMC5815069; doi:10.1093/femsyr/fow053)
Supplement: Supplementary Data [file fow053_supplementary_data.zip › Suplementary figure legends.docx]

**SUPPLEMENTARY FIGURE LEGENDS**

**Figure S1.** ITS PCR and RFLP for group identification. Group I strains yield a 3 band pattern and Group II strains a 4 band profile. Strain list: lane 1, GeneRuler^TM^ 1 kb DNA Ladder, Thermo Scientific.

**Figure S2.** Karyotyping of the Group II strains WS34/70 and A15, Group I strains A11, A58, A231 and A203, ale strains A60 and A115 and *S. eubayanus* C902. First and last lane, *S. cerevisiae* YNN295 used for molecular weight calibration.

**Figure S3.** PFGE filter probed with *ScMALx1*. The filter was exposed for 2 days. Position and size of relevant chromosomes based in the reference *S. cerevisiae* YNN295 is shown.

**Figure S4.** PFGE filter probed with *SeMALx1*. The filter was exposed for 2 days. *S. eubayanus* chromosomes were identified based in the relative position to the reference *S. cerevisiae* YNN295. Sizes of *S. eubayanus* chromosomes in the strains A220, A231 and A203 are described in van den Broek *et al*. (2015).

**Figure S5.** PFGE filter probed with *ScAGT1*. The filter was exposed for 2 days. Position and size of relevant chromosomes based in the reference *S. cerevisiae* YNN295 is shown.

**Figure S6.** PFGE filter probed with *SeAGT1*. The filter was exposed for 2 days. Position and size of relevant chromosomes based in the reference *S. cerevisiae* YNN295 is shown.

**Figure S7.** PFGE filter probed with *MPHx*. The filter was exposed for 2 days. Position and size of relevant chromosomes based in the reference *S. cerevisiae* YNN295 is shown.

**Figure S8.** PFGE filter probed with *MTT1*. The filter was exposed for 2 days. Position and size of relevant chromosomes based in the reference *S. cerevisiae* YNN295 is shown.
