# Supplementary material for: Maltose and maltotriose utilisation by group I strains of the hybrid lager yeast Saccharomyces pastorianus
Source: FEMS Yeast Res. 2016 Jun 30;16(5):fow053. doi: 10.1093/femsyr/fow053 (PMC5815069; doi:10.1093/femsyr/fow053)
Supplement: Supplementary Data [file fow053_supplementary_data.zip › Supplementary tables.docx]

**Table S1.** Oligonucleotides used for quantitative PCR analysis

| Transporter | Sequence | Amplicon size (b) |
| --- | --- | --- |
| *ScMALx1* | GGTATTAACAGGAGAAGAACGAG | 282 |
|  | TGTCTGAACATCCTAAACCACC |  |
| *SeMALx1* | GTATATCCCATGTCCGAGTCTG | 147 |
|  | GTCTCCTATGCCTCTGAAATCTG |  |
| *MPHx* | CGACAGCATTGAATACATATCCC | 204 |
|  | ATATAACATAAGGTCAGCCCGA |  |
| *ScAGT1* | GTCCATATTAGTGTCTACTACCCT | 128 |
|  | GTAATTTCGTAAGAACCCTCCC |  |
| *SeAGT1* | CTCGCTAATTCAATACTGTCTTGG | 278 |
|  | ATGGTCTTGGTCCTTAATTCTG |  |
| *MTT1* | TTGTTTATGTTGGGTCGGTC | 201 |
|  | CAATCCAAATGCGTAAAGGTC |  |

**Table S2**. Oligonucleotide probes used for southern hybridization

| Transporter | Sequence | Sequence detected (b) | Ref. |
| --- | --- | --- | --- |
| *ScMALx1* | GGTTTCTGGTAAATCGACAACAGCCCAAGCTAA | 1615-1647 | Gibson *et al*., 2013b |
| *SeMALx1* | AGTAACCTATTAGTGACGCACCACAAACAGTTTG | 1117-1150 | This work |
| *MPHx* | TGGAAGCTTCGATATCTCCCTTGGGGTCGTTATGAGAGACG | 1754-1794 | Gibson *et al*., 2013b |
| *ScAGT1* | ATGCTAAAGGGCAAACTTCCGAAGCATAAGTAACAG | 683-718 | This work |
| *SeAGT1* | TTCGTAGGAGCCTTCCGCATTCATAGTCCCA | 420-450 | Gibson *et al*., 2013b |
| *MTT1* | ACTGTTTGTATAGCCAATCCAAATGCGTAAAGGTCAAAC | 1278-1316 | Gibson *et al*., 2013b |

**Table S3.** Relative copy number of transporter genes as determined by quantitative PCR

|  | Transporter genes | | | | | |
| --- | --- | --- | --- | --- | --- | --- |
| Strain | ScMALx1 | SeMALx1 | ScAGT1 | SeAGT1 | MPHx | MTT1 |
| A11 | 3.0 (±0.28) | 3.2 (±0.31) | - | 0.7 (±0.74)* | - | - |
| A58 | 0.6 (±0.12) | 4.3 (±0.18) | 1.0 (±0.16) | 0.7 (±0.22) | - | 4.6 (±1.41) |
| A231 | 1.7 (±0.13) | 3.6 (±1.19) | 1.0 (±0.05) | 1.1 (± 1.81) | - | 2.6 (±0.73) |
| A203 | 2.6 (±0.09) | 2.7 (±0.10) | 1.0 (±0.41) | 1.0 (± 0.07) | - | 2.7 (±0.04) |
| A15 | 3.0 (±0.17) | 4.0 (±0.23) | 1.0 (±0.12) | 1.0 (± 0.02) | 1.0 (±0.54) | 1.6 (±0.12) |
| A220 | 3.9 (±0.74) | 5.3 (± 0.61) | 1.0 (±0.11) | 0.7 (± 1.21) | 1.1 (±0.72) | 1.8 (±0.92) |
| A60 | 4.3 (±1.01) | - | 1.0 (±0.34) | - | - | - |
| A115 | 3.8 (±0.77) | - | 1.0 (±0.17) | - | 1.3 (±0.34) | 1.4 (±0.53) |
| C902 | - | 2.8 (± 0.24) | - | - | - | - |

*SeAGT1 was not detected in the strain A11 by southern hybridization
