# Supplementary figures and images for: Maltose and maltotriose utilisation by group I strains of the hybrid lager yeast Saccharomyces pastorianus
Source: FEMS Yeast Res. 2016 Jun 30;16(5):fow053. doi: 10.1093/femsyr/fow053 (PMC5815069; doi:10.1093/femsyr/fow053)

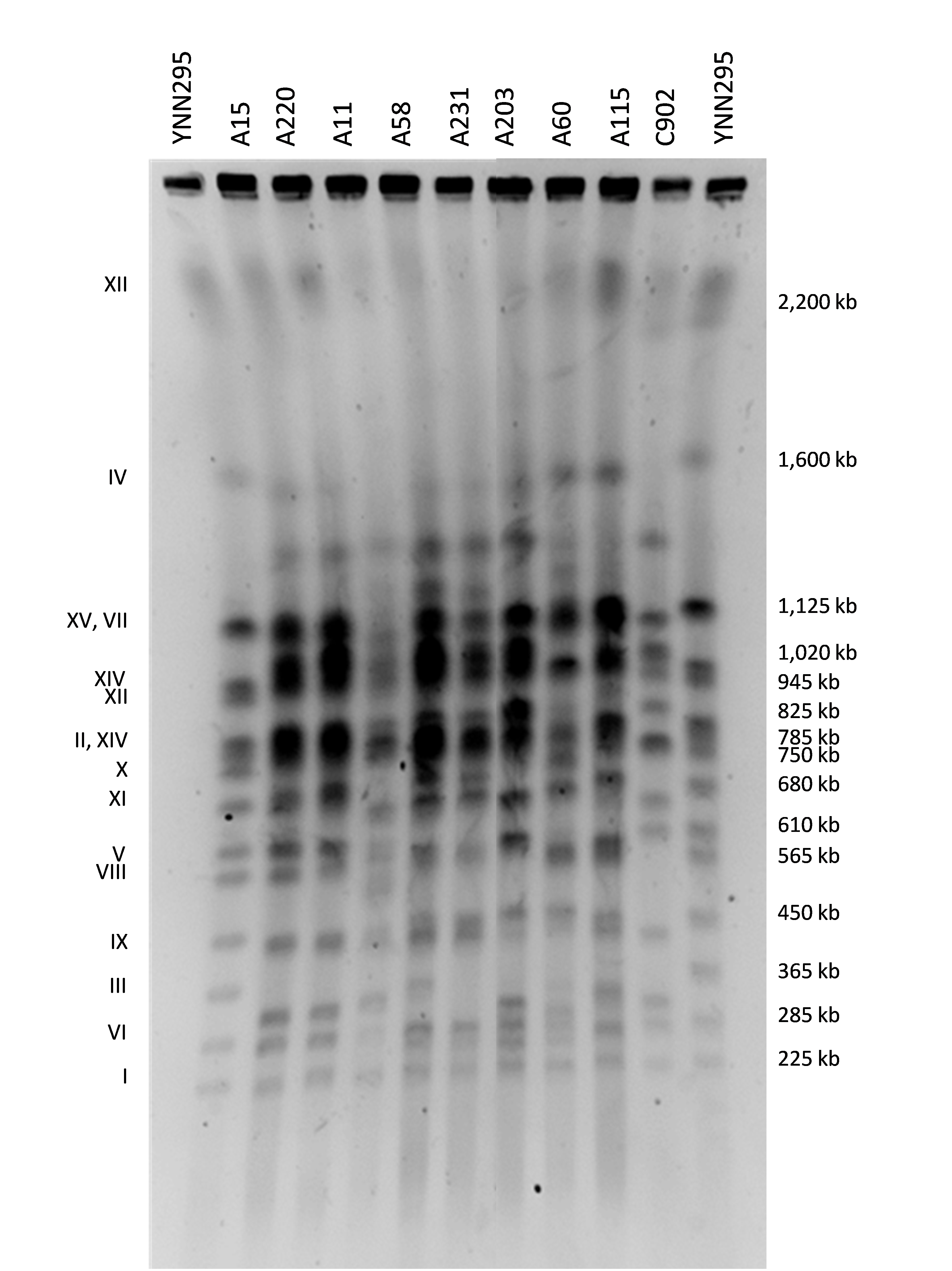

Supplement: Supplementary Data [file fow053_supplementary_data.zip › Figure S2.jpg]

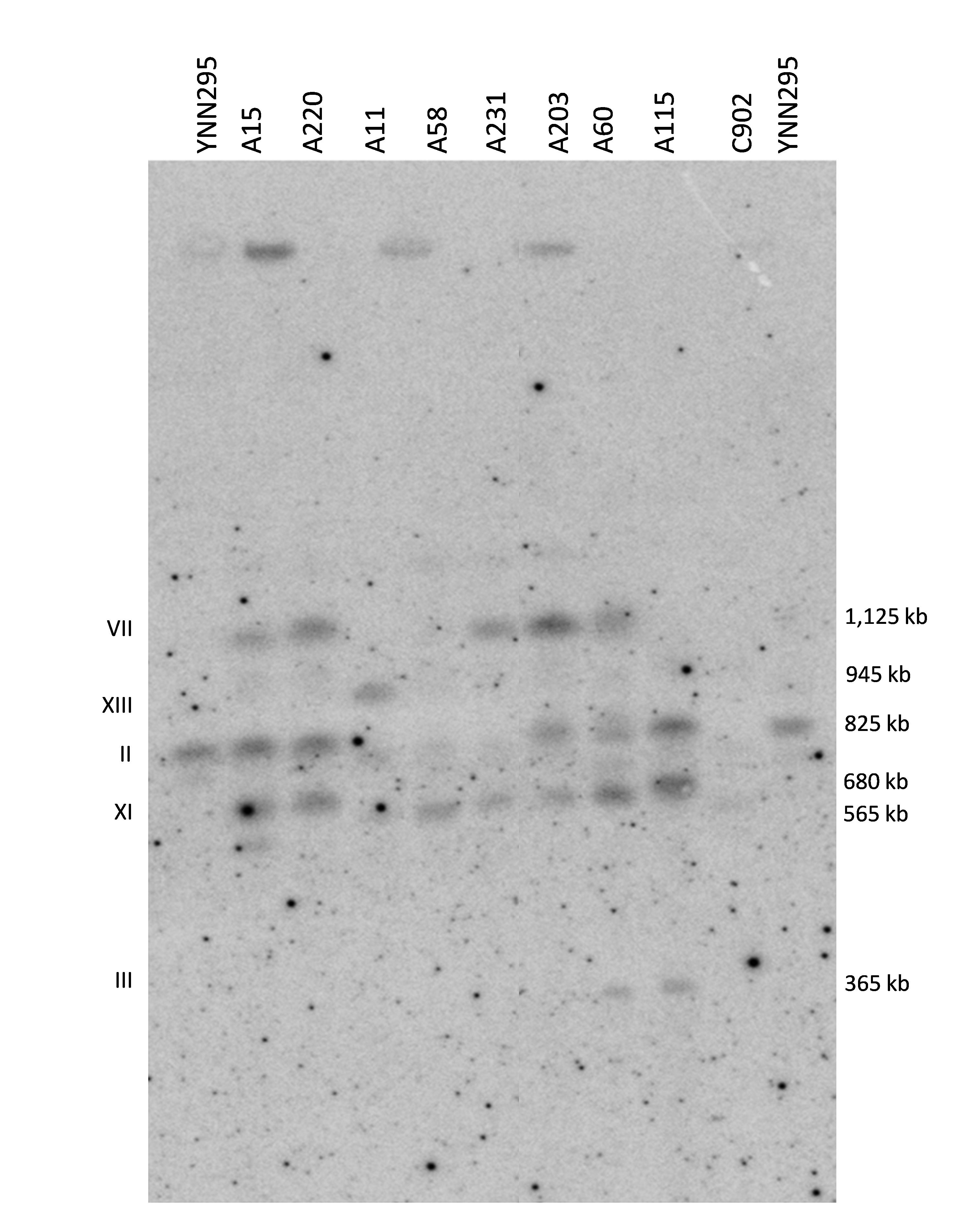

Supplement: Supplementary Data [file fow053_supplementary_data.zip › Figure S3.jpg]

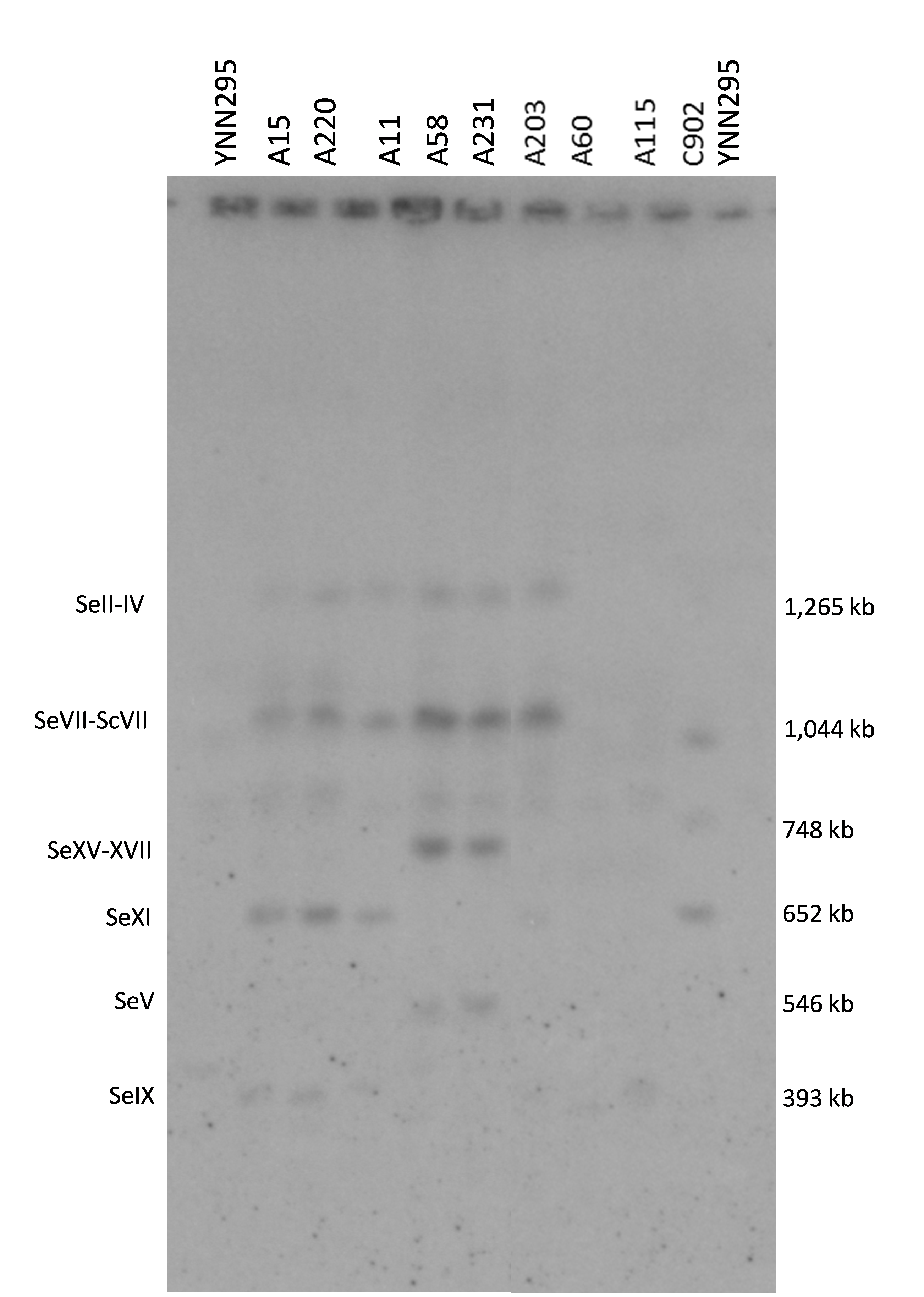

Supplement: Supplementary Data [file fow053_supplementary_data.zip › Figure S4.jpg]

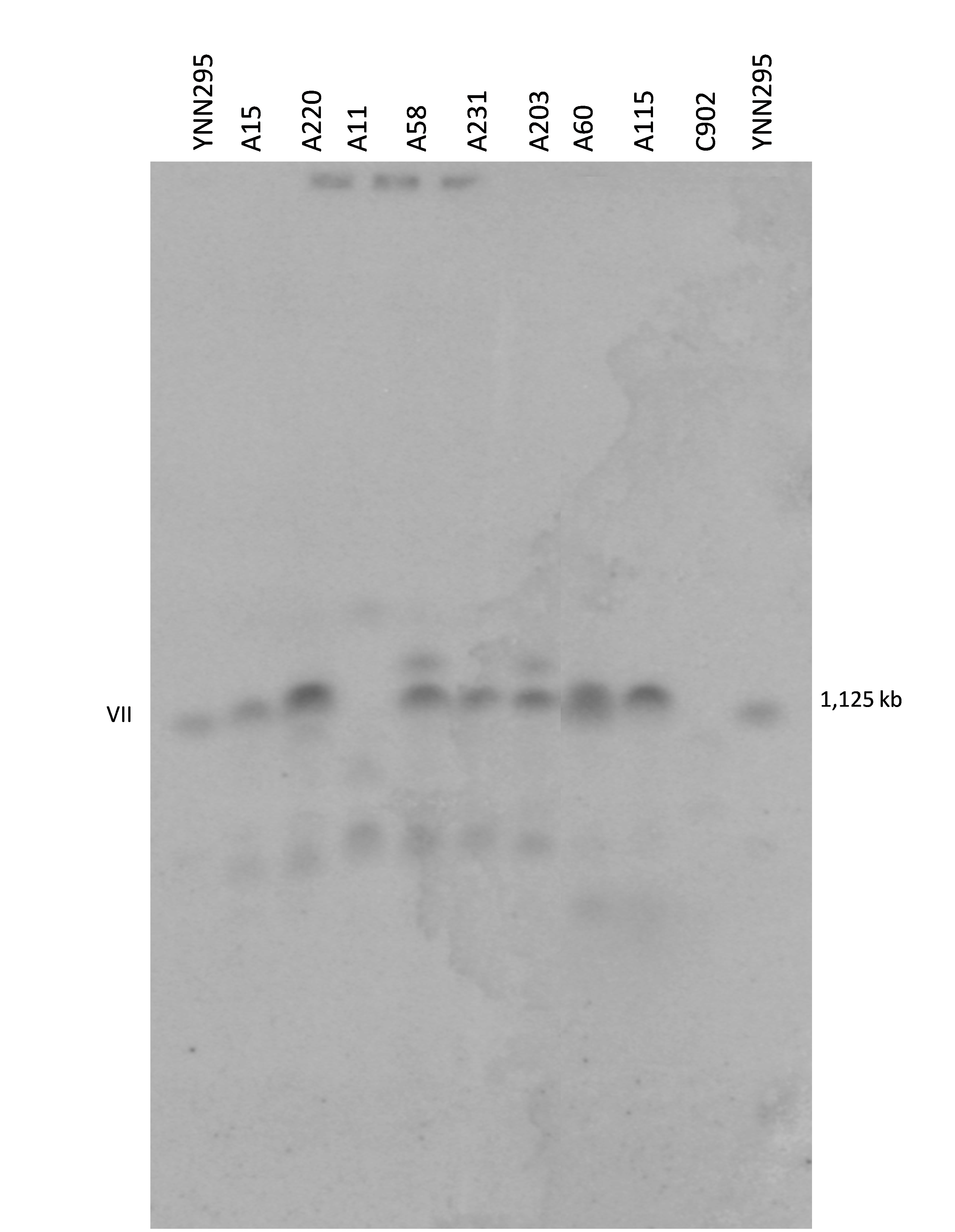

Supplement: Supplementary Data [file fow053_supplementary_data.zip › Figure S5.jpg]

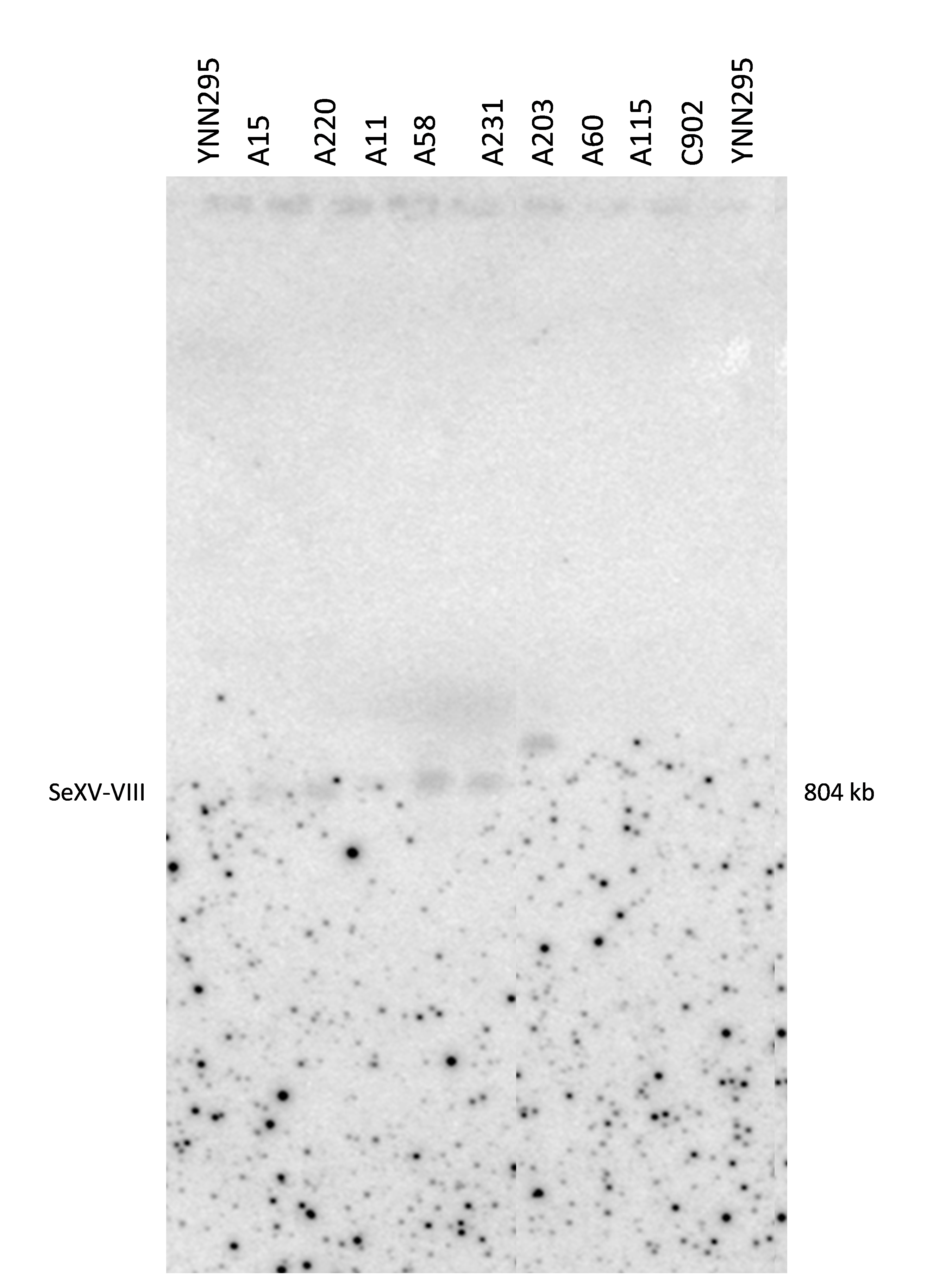

Supplement: Supplementary Data [file fow053_supplementary_data.zip › Figure S6.jpg]

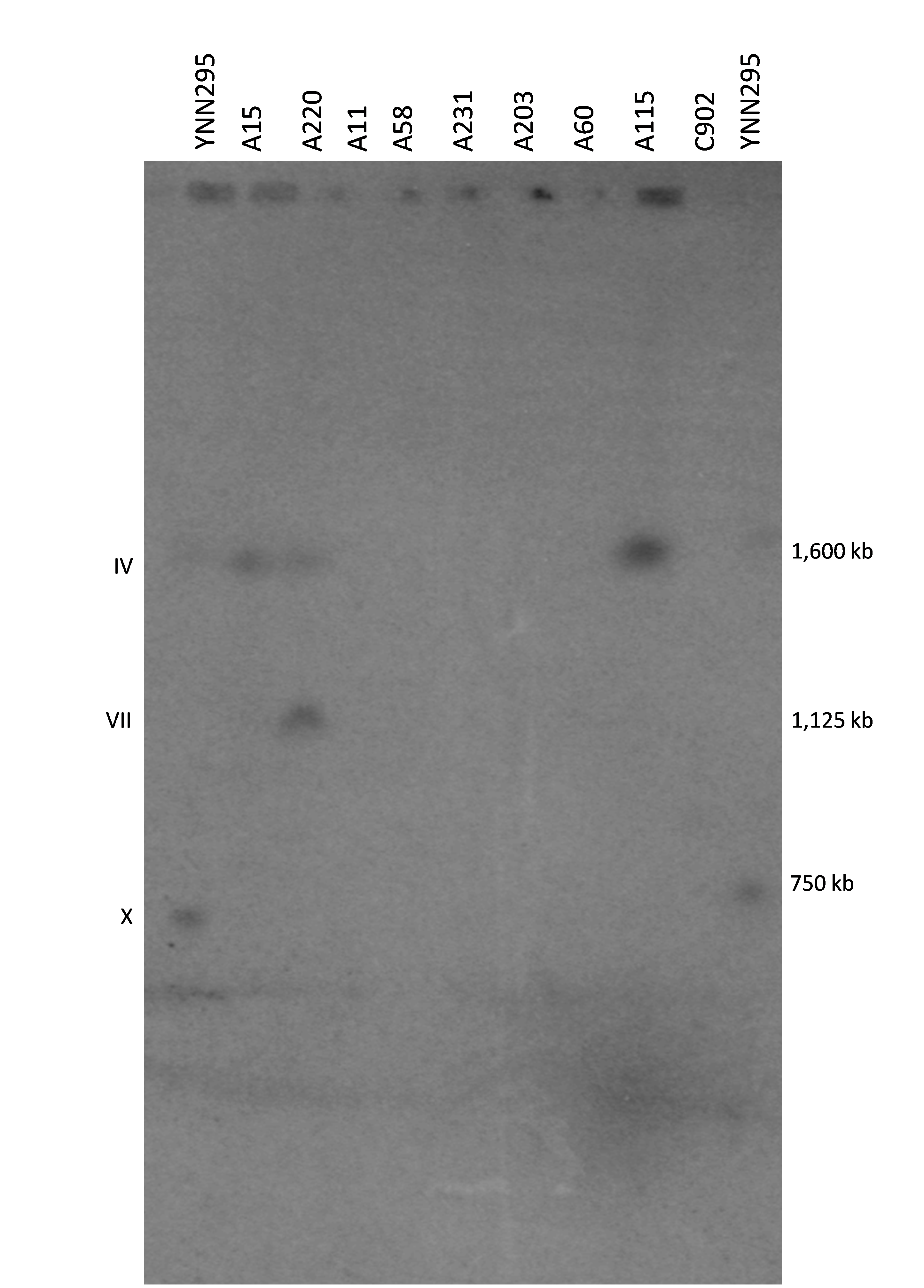

Supplement: Supplementary Data [file fow053_supplementary_data.zip › Figure S7.jpg]

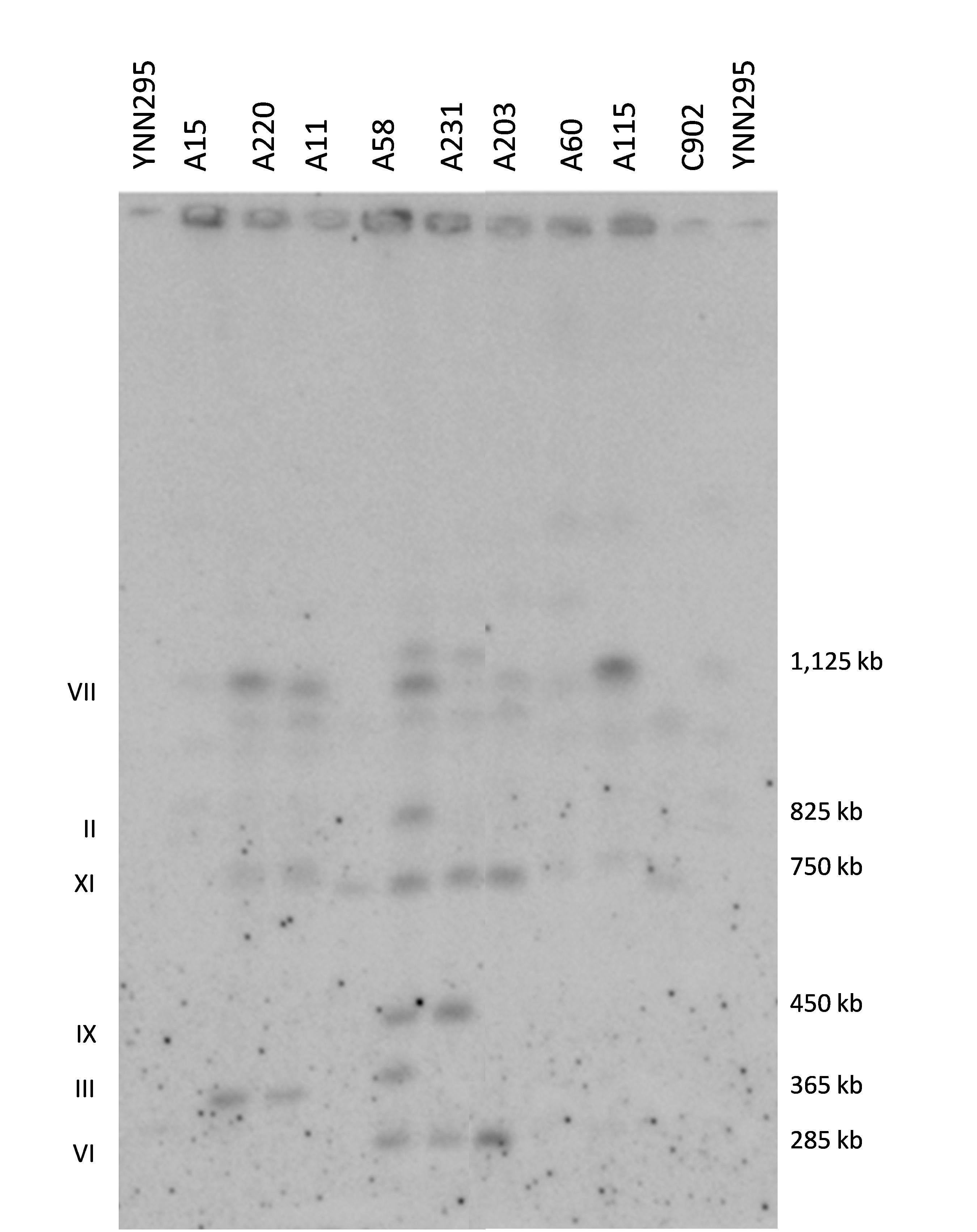

Supplement: Supplementary Data [file fow053_supplementary_data.zip › Figure S8.jpg]

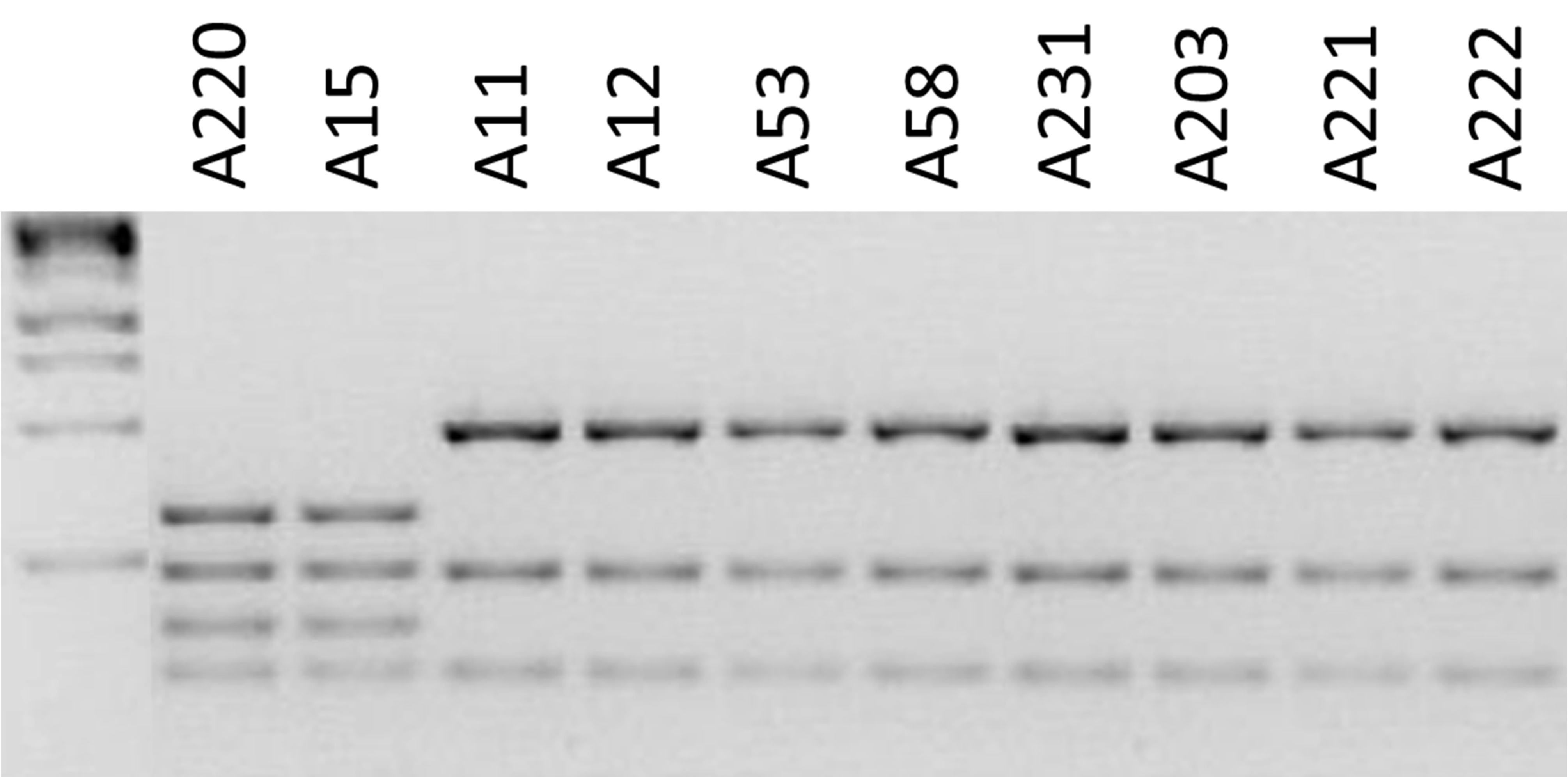

Supplement: Supplementary Data [file fow053_supplementary_data.zip › Figure_S1.jpeg]
